# ZnO–ferromagnetic metal vertically aligned nanocomposite thin films for magnetic, optical and acoustic metamaterials†

**DOI:** 10.1039/d2na00444e

**Published:** 2022-11-22

**Authors:** Robynne L. Paldi, Matias Kalaswad, Juanjuan Lu, James P. Barnard, Nicholas A. Richter, Mengwei Si, Nirali A. Bhatt, Peide D. Ye, Raktim Sarma, Aleem Siddiqui, Jijie Huang, Xinghang Zhang, Haiyan Wang

**Affiliations:** School of Materials Engineering, Purdue University West Lafayette Indiana 47907 USA hwang00@purdue.edu; School of Electrical and Computer Engineering, Purdue University West Lafayette Indiana 47907 USA; Birck Nanotechnology Center, Purdue University West Lafayette 47907 USA; Sandia National Laboratories New Mexico USA; School of Materials, Sun Yat-sen University Guangzhou Guangdong 510275 China

## Abstract

Magnetoacoustic waves generated in piezoelectric and ferromagnetic coupled nanocomposite films through magnetically driven surface acoustic waves present great promise of loss-less data transmission. In this work, ferromagnetic metals of Ni, Co and Co_*x*_Ni_1−*x*_ are coupled with a piezoelectric ZnO matrix in a vertically-aligned nanocomposite (VAN) thin film platform. Oxidation was found to occur in the cases of ZnO–Co, forming a ZnO–CoO VAN, while only very minor oxidation was found in the case of ZnO–Ni VAN. An alloy approach of Co_*x*_Ni_1−*x*_ has been explored to overcome the oxidation during growth. Detailed microstructural analysis reveals limited oxidation of both metals and distinct phase separation between the ZnO and the metallic phases. Highly anisotropic properties including anisotropic ferromagnetic properties and hyperbolic dielectric functions are found in the ZnO–Ni and ZnO–Co_*x*_Ni_1−*x*_ systems. The magnetic metal–ZnO-based hybrid metamaterials in this report present great potential in coupling of optical, magnetic, and piezoelectric properties towards future magnetoacoustic wave devices.

## Abstract 2

Magnetoacoustic waves generated in piezoelectric and ferromagnetic coupled nanocomposite films through magnetically driven surface acoustic waves present great promise of loss-less data transmissions.

## Graphical abstract

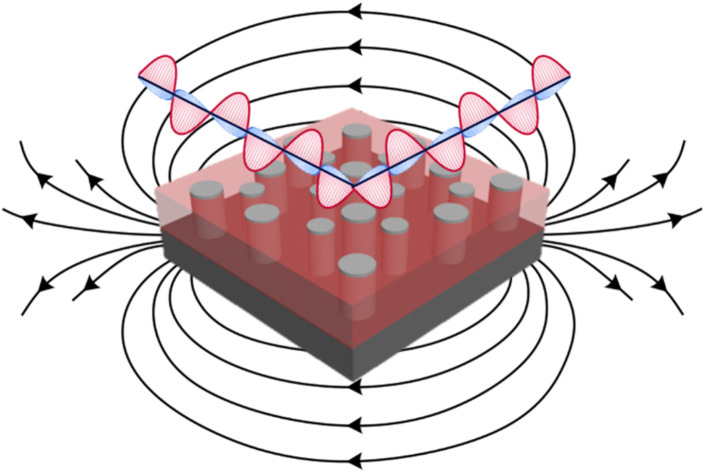

## Introduction

Recently, magnetoacoustic wave devices have been realized as an avenue for low-loss data transmission and next generation on-chip communication.^1^ In the seminal work, interdigitated ferromagnetic Ni film was grown on LiNbO_3_ substrates and magnetoacoustic waves were generated through the magnetoelastic effect over millimeter distances.^1^ The magnetoelastic effect is a change of magnetic field in response to a mechanical stress, or *vice versa*. In the Ni–LiNbO_3_ coupled device, ferromagnetic properties in the Ni film were tuned by strain in the piezoelectric LiNbO_3_ driven by surface acoustic waves. The hybridized piezoelectric-ferromagnetic Ni–LiNbO_3_ platform^1^ was used to drive magnetoacoustic waves with high amplitude over long distances, overcoming the magnetic dampening effects usually hindering application of ferromagnets by use of the magnetoelastic effect.^1^ Magnetoacoustic waves have also been realized in dipolar-coupled bilayer ferromagnetic thin films grown on LiNbO_3_ substrates towards application in acoustic resonators.^2^ In this case, non-reciprocity was also measured, demonstrating breaking of the time reversal symmetry.^3,4^ Breakage of time reversal symmetry implies the existence of certain topological states^5^ existing in these piezoelectric–ferromagnetic coupled composites and could prove as effective approaches for realizing topological phononic metamaterials which could allow for loss-less transmission of elastic waves in all directions.^6^

Very recently, the oxide–metal vertically-aligned nanocomposite (VAN) platform has gained attention as an effective design approach for hybrid plasmonic metamaterials.^7–12^ Moreover, anisotropic magnetic and electrical conductivity can be designed into the platform.^13,14^ In the oxide–metal VANs, two immiscible phases are self-assembled through co-deposition resulting in the epitaxial growth of nanopillars of the metallic phase in the oxide matrix. The benefits of the VAN are the highly anisotropic microstructure and physical properties in the systems, very uniformly distributed nanopillars, the high-quality and tailorable epitaxial growth, and the ability to couple magnetic, optical, and electrical multifunctionalities.^14–17^

**Fig. 1:**
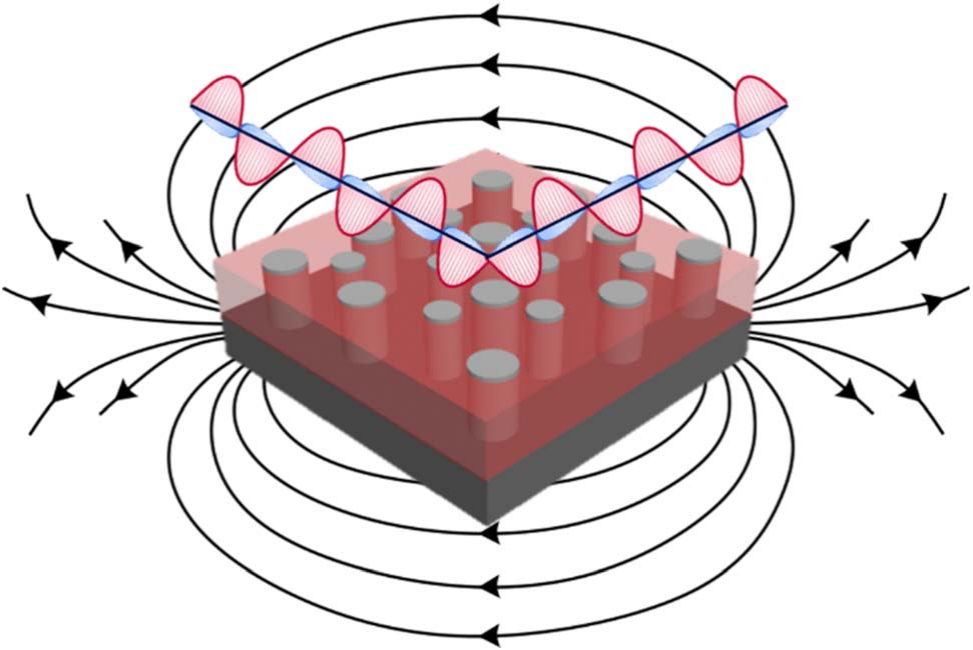
Ferromagnetic metal and ZnO nanocomposite design with magnetic and optical response.

In this letter, piezoelectric ZnO matrix is grown with ferromagnetic metals of Ni, Co, and their nanoalloy Co_*x*_Ni_1−*x*_. ZnO is selected as the matrix because of its strong dielectric and piezoelectric response.^18–20^ Co and Ni are selected for the pillar phase owing to their strong ferromagnetic response and potential plasmonic properties.^21–23^ The schematic diagram in Fig. 1 depicts the designs of the nanocomposite grown in this report, including both hyperbolic metamaterial responses due to plasmonic ferromagnetic pillars of Ni and Co embedded into dielectric matrix and for the potential magnetoacoustic wave propagation due to piezoelectric–ferromagnetic coupling. The issues to overcome include the potential oxidation of Ni and Co, in which the nanoalloy Co_*x*_Ni_1−*x*_ is proposed to resolve any oxidation issues. Nanocomposite thin films of ZnO–Ni, ZnO–Co, and ZnO–Co_*x*_Ni_1−*x*_ were grown and their morphology was characterized through X-ray diffraction and transmission electron microscopy. Optical properties are characterized through spectroscopic ellipsometry, and magnetic properties were measured with MPMS SQUID to explore the anisotropic nature of these ZnO–ferromagnetic metal hybrid metamaterials.

## Discussion/results

**Fig. 2:**
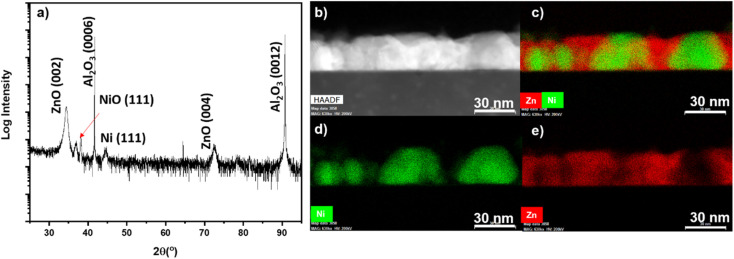
Microstructural characterization of the ZnO–Ni nanocomposite. (a) 2*θ* scan. (b) Cross-section STEM image. (c) Combined EDS-mapping. (d) EDS-mapping for Ni. (e) EDS-mapping for Zn element.

Ferromagnetic metals of Ni, Co, and their nanoalloy Co_*x*_Ni_1−*x*_ were combined with dielectric/piezoelectric ZnO matrix to form vertically-aligned nanocomposite thin films. Films were grown on c-cut sapphire substrates. The difficulty in growing ferromagnetic metal/oxide composites come from the tendency of Co and Ni to easily oxidize during the oxide growth. First, vacuum deposition was explored to minimize the metal oxidation during growth. Microstructural characterization of the ZnO–Ni nanocomposite is depicted in Fig. 2. The out-of-plane *θ*–2*θ* scan is shown in Fig. 2a, of which peaks from ZnO (0002) and Ni (111) were measured. From the scan, minor oxide peaks of NiO_*x*_ and a weak NiO (111) peak appeared, indicating minor oxidation of the Ni phase. By comparing the Ni (111) and NiO (111) peaks, the film can be considered as a primary ZnO–Ni VAN system. A scanning transmission electron microscopy (STEM) micrograph of the cross-section of ZnO–Ni VAN is shown in Fig. 2b. STEM contrast is derived from the atomic number, where contrast is ∼*Z*^1.4^, *Z*_Ni_ = 28 and *Z*_Zn_ = 30, leading to little contrast in the STEM image between ZnO and Ni. Energy dispersive (EDS) elemental mapping was performed in Fig. 2c–e. The combined map of Zn and Ni is demonstrated in Fig. 2c, showing more clearly the morphology of the ZnO–Ni VAN as compared with the STEM image in Fig. 2b, Ni grows as wide pillars embedded into the ZnO matrix. Based on the STEM images, the dimensions of Ni nanopillars are summarized in Table S1.† According to Table S1,† these wide Ni pillars grow with an average height of 27.7 ± 2.2 nm and width of 43.6 ± 1.8 nm, while the smaller particle-like growths were measured to have an average height of 18.1 ± 2.2 nm and width of 18.1 ± 5.7 nm. The individual maps for Ni and Zn are shown in Fig. 2d and e, respectively, and not much overlap is shown as Ni and ZnO grow as separate phases without much interdiffusion.

Similarly, ZnO–Co nanocomposite was explored in this work and the results of the microstructure characterization are shown in Fig. S1.† For ZnO–Co composite, two different substrate temperatures of 500 °C and 700 °C were explored. The XRD 2*θ* for 500 °C and 700 °C are shown in Fig. S1a and b,† respectively. At high temperature growth of 700° in Fig. S1b,† the film grows with ZnO (0002) and CoO (111) out-of plane orientation and no Co peaks were found, indicating the ZnO–Co composite actually grows as a ZnO–CoO oxide–oxide nanocomposite. The temperature was lowered to 500 °C in Fig. S1a† to explore if the high substrate temperature of 700 °C was leading to oxidation due to diffusion, but in the 500 °C 2*θ* the same peak for CoO (111) is found and no Co peak were identified. This serious oxidation of Co phase is attributed to the lower oxidation activation energy and faster oxidation rate of Co compared with Ni.^24^ A cross-section STEM image of the 500 °C sample is shown in Fig. S1c,† again with little contrast due to the similar atomic number of *Z*_Zn_ = 30 and *Z*_Co_ = 27. EDS-mapping of the ZnO–Co film grown at 500 °C was performed and shown in Fig. S1d–f.† The combined map in Fig. S1d† shows the morphology, with Co rich peaks growing inside ZnO matrix. Individual maps for Zn and Co are shown in Fig. S1e and f,† respectively. From the EDS-mapping, there is some interdiffusion between the Zn and Co areas, which is consistent with the literature; *i.e.*, Co diffuses easily into ZnO to form phases of Co-doped ZnO.^25–28^ The Co pillars were observed to grow substantially larger than those in the Ni-doped ZnO, with an average height and width of 134.0 ± 2.4 nm and 31.5 ± 4.6 nm, respectively, in the 500 °C sample. The microstructure STEM cross-section image of the ZnO–Co composite grown at 700 °C is shown in Fig. S1g† and the EDS mappings are depicted in Fig. S1h and i.† At higher temperature Co rich pillars grow in the ZnO matrix with some interdiffusion between the Co and ZnO, similar to the 500 °C growth. Since CoO is an antiferromagnetic material, the oxidation of Co phase in these films is a major hurdle for a strong ferromagnetic response in this system.^29^

**Fig. 3:**
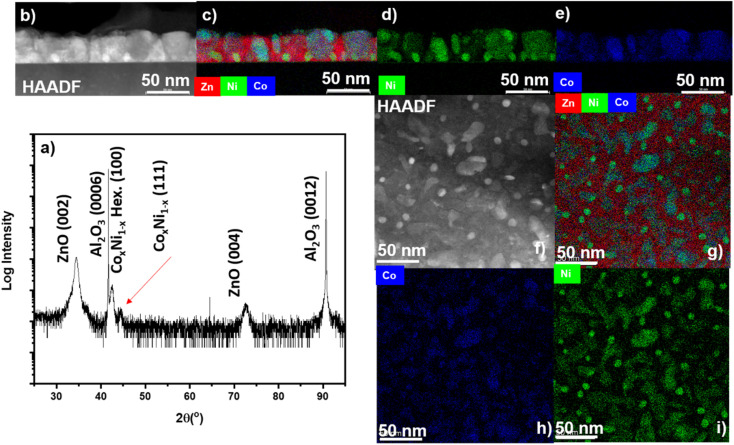
Microstructural characterization of the ZnO–Co_*x*_Ni_1−*x*_ nanocomposite. (a) 2*θ* scan. (b) cross-section STEM. (c) Combined EDS-mapping. (d) EDS mapping Ni element. (e) EDS-mapping element. (f) In-plane STEM image. (g) Combined EDS-mapping plan-view. (h) EDS-mapping Co. (i) EDS-mapping Ni element.

To overcome the oxidation issues faced in a single-phase ferromagnetic metal co-grown with ZnO, a nanoalloy approach was adopted. Recently, a Au_*x*_Ag_1−*x*_ nanoalloy was utilized in the ZnO VAN system to overcome particle-in-matrix morphology, oxidation issues in the ZnO–Ag system, and improve optical properties.^8^ Oxide–metal VANs have shown advantages in solving the oxidation problem because of their unique morphologies. In the previously reported ZnO–Au_*x*_Ag_1−*x*_ system, the uniform intermixing of Au and Ag phases impedes the diffusion of oxygen from the ZnO–metal interfaces. There are also plenty of three-phase oxide–metal VAN systems that have core–shell-structure nanopillars, which prevents the oxidation of metals. Based on the above-described results, a nanoalloy of Co_*x*_Ni_1−*x*_ was designed to form ZnO–Co_*x*_Ni_1−*x*_ VAN. It is hypothesized that the alloy formation is preferential over diffusion into the ZnO matrix or oxidation. Both Ni and Co are located next to each other on the periodic table (*Z*_Ni_ = 28 and *Z*_Co_ = 27) and have a very similar atomic radius, with *r*_Co_ = 0.135 nm and *r*_Ni_ = 0.135 nm,^30^ making it easy for solid solution alloying. Moreover, it is possible for Co to form a metastable face-centered cubic lattice (FCC) with similar structure to the Ni FCC lattice, with lattice parameter *a*_Co_ = 3.54 nm and *a*_Ni_ = 3.52 nm.^31^ Ni can also form a metastable hexagonal close-pack phase with similar lattice parameter as stable HCP Co.^32^ The results of microstructure characterization of ZnO–Co_*x*_Ni_1−*x*_ nanocomposite are shown in Fig. 3. The XRD *θ*–2*θ* scan are presented in Fig. 3a and peaks from ZnO (0002) and the nanoalloy are shown. Interestingly, two peaks for the alloy were measured, one corresponding to Co_*x*_Ni_1−*x*_ (100) and another to Co_*x*_Ni_1−*x*_ (111). The (100) peak may correspond to a Co-rich hexagonal phase and the (111) peak may correspond to a Ni-rich cubic phase. The cross-section STEM is shown in Fig. 3b. It has a similar issue as that of both ZnO–Ni and ZnO–Co composites, *i.e.*, very little contrast due to the similar atomic number of Zn, Co, and Ni. The EDS-mapping for cross-section is depicted in Fig. 3c–e, including combined, Co, and Ni and the EDS mapping of the Zn matrix cross-section is shown in Fig. S2a.† From the EDS-mapping, it is interesting to note that the alloy formation is not homogeneous. Ni-rich particles form near the sapphire substrate and some pillars appear to be Co-rich. The Ni-rich particles were measured based on the STEM images shown in Fig. 3, and they are found to have an average height and width of 9.8 ± 3.2 nm and 8.8 ± 3.9 nm, respectively, while the Co-rich pillars were measured to have an average height and width of 28.8 ± 9.9 nm and 25.6 ± 13.0 nm, respectively. The composition uniformity improvement as well as morphology tuning is promising in the future studies by changing the deposition parameters such as deposition temperature, laser energy, *etc.* A plan-view STEM is shown in Fig. 3f with plan-view EDS mapping depicted in Fig. 3g–i for combined, Ni, and Co; the Zn plan-view EDS-mapping is shown in Fig. S2b.† In the plan-view, Ni-rich particles with Co-rich areas forming with much larger and irregular shape. The delineation between the alloy phase and ZnO matrix is evidenced in the Zn plan-view EDX mapping in Fig. S2b.† Based on the EDS mapping data, the overall ratio between Co and Ni is determined to be around 1.975, which is the alloy composition of Ni_67_Co_33_. The target used for the deposition has a ratio of Ni/Co = 1 : 1. The difference between the target and film composition is possibly due to the laser ablation rate difference between Ni and Co. From the view of PLD deposition, the tuning of the Ni : Co ratio is highly possible by further manipulating the background atmosphere, target composition, as well as laser energy, laser frequency, *etc.*

**Fig. 4:**
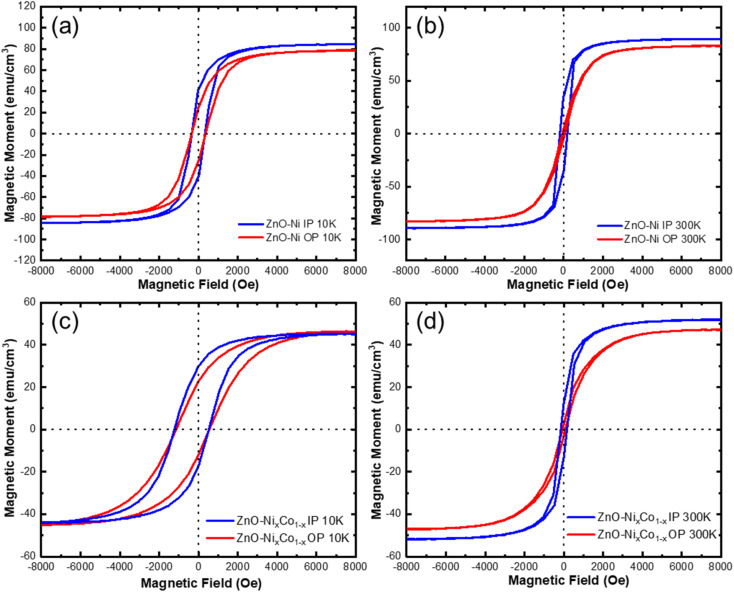
Anisotropic magnetic measurement. (a) ZnO–Ni at 10 K, (b) ZnO–Ni at 300 K, (c) ZnO–Ni_*x*_Co_1−*x*_ at 10 K, (d) ZnO–Ni_*x*_Co_1−*x*_ at 300 K. Note that the diamagnetic responses from the non-magnetic holder and substrate were linearly subtracted from the original data using the slope calculated at high field (8000–10 000 Oe).

One of the main purposes of this report is to realize ferromagnetic properties in a piezoelectric and dielectric matrix. Anisotropic magnetic hysteresis loops were obtained for ZnO–Ni, ZnO–Co, and ZnO–Co_*x*_Ni_1−*x*_ nanocomposites by SQUID MPMS in both the in-plane (IP) and out-of-plane (OP) directions. It is possible to modify the *H*_c_ and *M*_s_ values in the thin film systems by changing the domain size, interfacial coupling, *etc.*,^33,34^ and it is unique in the VAN systems to significantly manipulate the magnetic anisotropy through shape anisotropy tuning.^18,35,36^ The ZnO–Ni and ZnO–Co_*x*_Ni_1−*x*_ were measured at 10 K and 300 K to investigate ferromagnetic properties at low and room temperatures and are presented in Fig. 4. Magnetic anisotropy is measured by applying a magnetic field parallel and perpendicular to detect the IP and OP magnetic response. The resulting hysteresis loops in Fig. 4 demonstrate a strong ferromagnetic response for both directions at 10 K and 300 K in ZnO–Ni and ZnO–Co_*x*_Ni_1−*x*_ nanocomposites. Some differences in *M*_s_ and *H*_c_ values from IP and OP directions were observed in all measurements. The structure-sensitive *H*_c_ is closely related to the dimensions and shape anisotropy of nanopillars as well as the crystallinity quality, since it represents the largest field required to reach saturation.^37^ The change in *M*_s_ compared to bulk metals, however, shows finite size effects when the surface/volume ratio of the material is large enough, such as in the thin film systems. This is due to the broken symmetry of the exchange bonds at the surfaces that introduces spin disorder.^34^ The ZnO–Ni composite measurement are shown in the top row at 10 K in Fig. 4a and 300 K in Fig. 4b. At 10 K, the hysteresis loop remains somewhat similar in the IP and OP directions with the magnetic moment *M*_s_ and the magnetic coercivity *H*_c_ having similar values. A minor exchange bias effect was observed for the 10 K data, suggesting the minor NiO growth in the film, as the coercive field values IP and OP are slightly shifted as observed in Table S2.† At 300 K, the shape anisotropy is much more dramatic with the IP direction having a much higher coercive field *H*_c_ while the exchange bias effect has been greatly reduced. Interestingly, the ZnO–Ni at room temperature behaves as a soft magnet in the out-of-plane direction and as a hard magnet in the IP direction. Comparing the *M*_s_ values with bulk values (485 emu cm^−3^), it is obvious that this film has a non-saturated magnetization even under a high magnetic field. This is a combined effect of the crystallinity quality and the spin disorder at the interfaces. From Fig. 4, all *M*–*H* loops present minor differences in *M*_s_ values (∼10%) from the IP and OP directions. Considering the VSM measurement errors such as instrumental SQUID drift, sample geometry deviation from the standard sample, and many other additive artifacts, the *M*_s_ differences are negligible. Instead the differences in *H*_c_ from IP and OP directions are obvious. This IP and OP magnetic anisotropy is a combined effect of shape anisotropy and magnetocrystalline anisotropy.^38^ Since the magnetic easy axis of Ni is along 〈111〉, consistent with the out-of-plane Ni growth orientation in the ZnO–Ni system, the anisotropy is evident and strongly correlated to the nanopillar aspect ratio (height/width). This clearly explains the obvious difference between IP and OP hysteresis loops at 300 K for the ZnO–Ni thin film. The shape-anisotropy seen in the magnetic data at room temperature could be attributed to the morphology in Fig. 2. Ni pillars have a greater width than height. Therefore, it could be that a thicker film could result in more out-of-plane magnetic anisotropy. It is interesting to note that when the temperature was decreased to 10 K, the IP and OP anisotropy became weaker compared to the 300 K results. Magnetic measurements were also performed on the ZnO–Co_*x*_Ni_1−*x*_ composite thin film at 10 K in Fig. 4c and 300 K in Fig. 4d. At 300 K, ZnO–Co_*x*_Ni_1−*x*_ demonstrated a comparatively stronger anisotropic magnetic behavior. But at 10 K, the anisotropy in *H*_c_ becomes weaker in this oxide–alloy sample, although the differences in the opening of the hysteresis loop is larger. It is interesting to note from the data in Table S2† that the ZnO–Co_*x*_Ni_1−*x*_ displays an obvious exchange bias, especially at the 10 K measurement than at 300 K. An enlarged view of the hysteresis can be seen in Fig. S3† with accompanying discussion and the magnetic measurement for ZnO–Co can be found in Fig. S4† with the accompanying discussion. *H*_c_, *M*_s_, and *M*_r_ values for all samples are summarized in Table S2.†

**Fig. 5:**
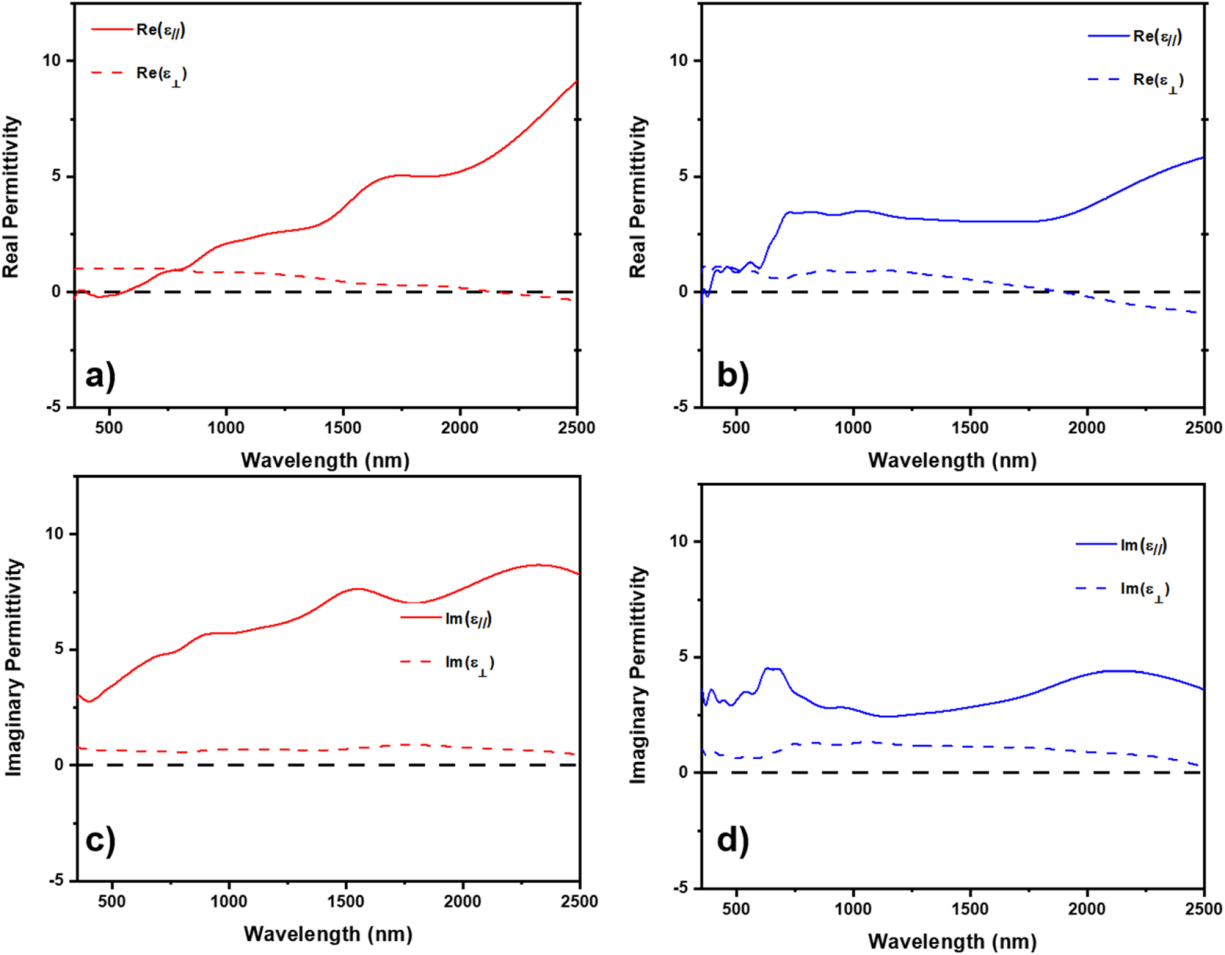
Measured and model real and imaginary permittivity. Real permittivity for (a) ZnO–Ni and (b) ZnO–Co_*x*_Ni_1−*x*_. Imaginary permittivity for (c) ZnO–Ni and (d) ZnO–Co_*x*_Ni_1−*x*_.

Both Ni and Co have been reported to have a plasmonic response in the near-infrared regime.^22,23,39,40^ The plasmon resonance is a discrete excitation of the electron cloud of a metal at the interface with a dielectric in response to light absorption.^41,42^ Due to the anisotropic morphology of the metallic inclusions when grown in the dielectric ZnO, it could be possible to realize hyperbolic dielectric response, *e.g.* behavior as a metal in one-direction and as a dielectric in the opposite direction. Both Ni and Co have been studied as low-loss alternatives for noble Au and Ag in plasmonic sensors and hyperbolic metamaterials.^39,43,44^ The complex dielectric function of ZnO–Ni and ZnO–Co_*x*_Ni_1−*x*_ composites were modeled through spectroscopic ellipsometry and are presented in Fig. 5. The dielectric model is based on psi and delta measurements in Fig. S5† and is a uniaxial B-spline with backside correction, and the optical axis is considered as along *z*-axis according to the unique uniaxial morphology of VANs. So *ε*_∥_ is the in-plane permittivity and *ε*_⊥_ is the permittivity along out-of-plane direction. As seen in Fig. 5, both ZnO–Ni and ZnO–Co_*x*_Ni_1−*x*_ demonstrate hyperbolic response in Fig. 5a and b, respectively. In Fig. 5a, ZnO–Ni has an epsilon-near zero (ENZ) permittivity point at around 2100 nm. The ZnO–Co_*x*_Ni_1−*x*_ film in Fig. 5b displayed its ENZ point near 1700 nm. Both composites are considered type I metallic hyperbolic metamaterial at wavelengths larger than the ENZ points, since in both materials, *ε*_∥_ > 0and *ε*_⊥_ < 0. The direction-dependent negative refraction of incident light enables applications such as selective optical filter, partial focusing of radiations, *etc.*^45–47^ Specifically, type I hyperbolic metamaterials have an isofrequency curve of two sheets and can support a high density of *k*-states as well as a broadly diverging density of photonic states,^48^ making them highly desirable in applications that require strong enhancement of spontaneous emission like subwavelength imaging techniques.^49,50^ It should also be noted that at lower wavelengths both systems demonstrate a characteristic of type II hyperbolic metamaterials, with *ε*_∥_ < 0 and *ε*_⊥_ > 0. For ZnO–Ni, this type II metamaterial region is at around 460–600 nm and for ZnO–Co_*x*_Ni_1−*x*_ this narrow region is at around 420–450 nm. Since those areas takes a small portion compared to the type I metamaterial region, both films are generally considered as type I metamaterial. The imaginary permittivity for ZnO–Ni and ZnO–Co_*x*_Ni_1−*x*_ in Fig. 5c and d, respectively, is related to the absorption in the film where lower values are more desirable for application. Absorption in the films in the work is relatively low, demonstrating the ferromagnetic/ZnO composite thin films as alternative low-loss metamaterials.

The advantage of the nanocomposites grown in this report are the coupled and tunable ferromagnetic, piezoelectric, and hyperbolic functionalities. The previous device structure of Ni–LiNbO_3_ (ref. 1) is limited in directionality due to the layered morphology. The nanocomposites in this report, due to the anisotropic morphology and properties of the ferromagnetic pillars embedded in the piezoelectric ZnO, are ideal to drive magnetoacoustic waves through the magnetoelastic effect in three dimensions by incorporation of a bottom electrode in a device architecture. Due to the hyperbolic optical response coupled with the magnetoacoustic wave generation, the ZnO-based ferromagnetic VAN presents great promises in realizing devices that can drive both phononic and photonic transmissions. Future work could include other ferromagnetic metals such as Fe and its alloys with Ni and Co, as well as other stronger piezoelectric matrices such as LiNbO_3_. Moreover, future work will be towards device integration and magnetoacoustic wave generation using these piezoelectric/ferromagnetic VAN systems.

## Conclusions

In this work, ferromagnetic metal pillars of Ni, Co, and Co_*x*_Ni_1−*x*_ nanoalloy were grown in a ZnO piezoelectric matrix. Oxidation was detected in both the ZnO–Ni and ZnO–Co system, with ZnO–Co forming a ZnO–CoO oxide–oxide vertically aligned-nanocomposite. The ZnO–Co_*x*_Ni_1−*x*_ VAN resulted in a morphology of Ni-rich and Co-rich inclusions embedded into ZnO. Magnetic measurements demonstrated obvious anisotropic ferromagnetic properties in both ZnO–Ni and ZnO–Co_*x*_Ni_1−*x*_ samples. The ZnO–Co_*x*_Ni_1−*x*_ also demonstrated an exchange bias effect at low temperatures. The ZnO–Co sample grown at 700 °C displayed ferromagnetic properties that may be the result of Co nanoparticles precipitating in the matrix due to the high temperature growth. Optical measurements confirm that ZnO–Ni and ZnO–Co_*x*_Ni_1−*x*_ systems have a hyperbolic dielectric response. The ZnO–ferromagnetic metal hybrid metamaterial systems demonstrated with tunable ferromagnetic and optical response are promising for future magneto-optic and acoustic applications.

## Experimental

### Thin film growth

Thin films of ZnO–Co, ZnO–Ni, and ZnO–Co_*x*_Ni_1−*x*_ were grown through the pulsed laser deposition method on c-cut sapphire (0001) substrates. ZnO–Ni and ZnO–Co_*x*_Ni_1−*x*_ were grown at 500 °C and ZnO–Co was grown at both 500 °C and 700 °C. A KrF excimer laser (Lambda Physik Complex Pro 205, *λ* = 248 nm) was used for all depositions with a laser energy of 420 mJ focused at an incident angle of 45°. The target–substrate distance was kept constant at 4.5 cm. All films were grown in a vacuum environment which was pumped down to ∼10^−6^ mtorr from atmospheric pressure after loading and after each deposition, were cooled at a rate of 15 °C min^−1^. The films were ablated from composite targets prepared through a conventional sintering method. The ZnO–Ni and ZnO–Co_*x*_Ni_1−*x*_ were consisting of 30% molar ratio of metal with the alloy mixed 1 : 1 Co : Ni. The ZnO–Co composite target contained 1 : 1 ZnO : Co composition.

### Microstructure characterization

Film morphology was characterized through XRD, TEM, and STEM coupled with EDS-mapping. XRD scans of *θ*–2*θ* were conducted using a Panalytical X'Pert X-ray diffractometer wit Cu K_α_ radiation. Bright field TEM, STEM, SAED patterns and EDS-mapping was performed in a FEI Talos F200X TEM. Samples for electron microscopy were prepared, for both cross-section and plan-view, *via* a standard grinding procedure which entails manual grinding, polishing, dimpling, and a final ion milling step to achieve electron transparency (PIPS 691 precision ion polishing system, 5 keV for cross-section and 4–4.5 keV for plan-view sample).

### Optical measurements

Ellipsometry measurements were carried out on an RC2 Spectroscopic ellipsometer (J.A. Woollam Company). Four angles 40°, 50°, 60°, and 70° were measured from a spectrum range of 210–2500 nm. Psi and delta data were obtained from ellipsometry measurements and then fit with a uniaxial model coupled with a B-spline model which were used to discern anisotropic permittivity properties of composite films. An agreeable mean square error (MSE) < 5 was obtained for all film models.

### Magnetic measurements

Magnetic property measurements were carried out on the MPMS SQUID (Quantum design MPMS 3) system at both 10 K and 300 K using vibrating sample magnetometer (VSM) mode. A magnetic field of 10 000 Oe was applied perpendicular and parallel to the sample surface for measuring OP and IP responses. All obtained data were processed with a field remanence correction and a subtraction of the dielectric slope which was generated by diamagnetic substrate and sample holders. The sample geometry factors of each sample from both IP and OP measurement directions were calculated by sample geometry simulator (SGS) from QD Pharos Digital library, as demonstrated in Table S3.† Then the magnetic moment data was divided by its corresponding geometry factors before plotting the *M*–*H* loops.

## Conflicts of interest

There are no conflicts to declare.

## Supplementary Material

NA-005-D2NA00444E-s001

## References

[cit1] Casals B., Statuto N., Foerster M., Hernández-Mínguez A., Cichelero R., Manshausen P., Mandziak A., Aballe L., Hernàndez J. M., Macià F. (2020). Phys. Rev. Lett..

[cit2] Küβ M., Heigl M., Flacke L., Hörner A., Weiler M., Wixforth A., Albrecht M. (2021). Phys. Rev. Appl..

[cit3] Verba R., Tiberkevich V., Slavin A. (2019). Phys. Rev. Appl..

[cit4] Haldane F. D. M., Raghu S. (2008). Phys. Rev. Lett..

[cit5] Lee J. S., Richardella A., Fraleigh R. D., xing Liu C., Zhao W., Samarth N. (2018). npj Quantum Mater..

[cit6] Guo S., Chen J., Huang H., Wei Y., Tan Z., Feng L., Xie X. (2021). Mech. Syst. Signal Process..

[cit7] Li L., Sun L., Gomez-Diaz J. S., Hogan N. L., Lu P., Khatkhatay F., Zhang W., Jian J., Huang J., Su Q., Fan M., Jacob C., Li J., Zhang X., Jia Q., Sheldon M., Alù A., Li X., Wang H. (2016). Nano Lett..

[cit8] Paldi R. L., Wang X., Sun X., He Z., Qi Z., Zhang X., Wang H. (2020). Nano Lett..

[cit9] Paldi R. L., Sun X., Wang X., Zhang X., Wang H. (2020). ACS Omega.

[cit10] Lu J., Paldi R. L., Pachaury Y., Zhang D., Wang H., Kalaswad M., Sun X., Liu J., Phuah X. L., Zhang X., El-Azab A. A., Wang H. (2021). Mater. Today Nano.

[cit11] Paldi R. L., Qi Z., Misra S., Lu J., Sun X., Phuah X. L., Kalaswad M., Bischoff J., Branch D. W., Siddiqui A., Wang H. (2021). Adv. Photonics Res..

[cit12] Paldi R. L., Sun X., Phuah X. L., Lu J., Zhang X., Siddiqui A., Wang H. (2021). Nanoscale Adv..

[cit13] Zhang B., Fan M., Li L., Jian J., Huang J., Wang H. H., Kalaswad M., Wang H. H. (2018). Appl. Phys. Lett..

[cit14] Su Q., Zhang W., Lu P., Fang S., Khatkhatay F., Jian J., Li L., Chen F., Zhang X., Macmanus-Driscoll J. L., Chen A., Jia Q., Wang H. (2016). ACS Appl. Mater. Interfaces.

[cit15] Huang J., Jin T., Misra S., Wang H. H., Qi Z., Dai Y., Sun X., Li L., Okkema J., Chen H.-T. T., Lin P.-T. T., Zhang X., Wang H. H. (2018). Adv. Opt. Mater..

[cit16] Huang J., Wang X., Hogan N. L., Wu S., Lu P., Fan Z., Dai Y., Zeng B., Starko-Bowes R., Jian J., Wang H. H. H. H., Li L., Prasankumar R. P., Yarotski D., Sheldon M., Chen H.-T. T., Jacob Z., Zhang X., Wang H. H. H. H. (2018). Adv. Sci..

[cit17] Misra S., Li L., Jian J., Huang J., Wang X., Zemlyanov D., Jang J. W., Ribeiro F. H., Wang H. (2018). ACS Appl. Mater. Interfaces.

[cit18] Mousavi S. H., Khanikaev A. B., Wang Z. (2015). Nat. Commun..

[cit19] Opel M., Goennenwein S. T. B., Althammer M., Nielsen K. W., Karrer-Müller E. M., Bauer S., Senn K., Schwark C., Weier C., Güntherodt G., Beschoten B., Gross R. (2014). Phys. Status Solidi B.

[cit20] Janotti A., Van De Walle C. G. (2009). Rep. Prog. Phys..

[cit21] Reser B. I. (1999). J. Phys.: Condens. Matter.

[cit22] Yu A. Y. C., Donovan T. M., Spicer W. E. (1968). Phys. Rev..

[cit23] Ehrenreich H., Philipp H. R., Olechna D. J. (1963). Phys. Rev..

[cit24] Frederick S. F., Cornet I. (1955). J. Electrochem. Soc..

[cit25] Rode K., Anane A., Mattana R., Contour J. P., Durand O., LeBourgeois R. (2003). J. Appl. Phys..

[cit26] Echeverria E., Kaphle A., Austin A., Bastatas L., Hari P., McLlroy D. (2019). ACS Appl. Nano Mater..

[cit27] Kim J. H., Kim H., Kim D., Ihm Y. E., Choo W. K. (2002). J. Appl. Phys..

[cit28] Perry N. H., Mason T. O. (2013). J. Am. Ceram. Soc..

[cit29] Peng D. L., Sumiyama K., Hihara T., Yamamuro S., Konno T. J. (2000). Phys. Rev. B: Condens. Matter Mater. Phys..

[cit30] Slater J. C. (1964). J. Chem. Phys..

[cit31] Lizárraga R., Pan F., Bergqvist L., Holmström E., Gercsi Z., Vitos L. (2017). Sci. Rep..

[cit32] Jeon Y. T., Moon J. Y., Lee G. H., Park J., Chang Y. (2006). J. Phys. Chem. B.

[cit33] Dong L., Liu Y., Lu Y., Zhang L., Man N., Cao L., Ma K., An D., Lin J., Xu Y. J., Xu W. P., Bin Wu W., Yu S. H., Wen L. P. (2013). Adv. Funct. Mater..

[cit34] Crisan O., Angelakeris M., Flevaris N. K., Filoti G. (2003). J. Optoelectron. Adv. Mater..

[cit35] Huang J., Li L., Lu P., Qi Z., Sun X., Zhang X., Wang H. (2017). Nanoscale.

[cit36] Kalaswad M., Zhang B., Wang H., Wang X., Huang J., Wang H. (2020). Mater. Today Adv..

[cit37] Cullity B. D. and Graham C. D., Introduction to Magnetic Materials, John Wiley & Sons, Inc., Hoboken, New Jersey, 2009

[cit38] Zhang B., Huang J., Rutherford B. X. X., Lu P., Misra S., Kalaswad M., He Z., Gao X., Sun X., Li L., Wang H. (2020). Mater. Today Nano.

[cit39] Shukla S., Sharma N. K., Sajal V. (2016). Braz. J. Phys..

[cit40] Kaminskiene Z., Prosyevasa I., Stonkute J., Guobiene A. (2013). Acta Phys. Pol., A.

[cit41] Lee D., So S., Hu G. (2022). eLight.

[cit42] Mubeen S., Lee J., Lee W. R., Singh N., Stucky G. D., Moskovits M. (2014). ACS Nano.

[cit43] Boltasseva A., Atwater H. A. (2011). Science.

[cit44] Khurgin J. B. (2017). Philos. Trans. R. Soc., A.

[cit45] Cho H., Yang Y., Lee D., So S., Rho J. (2021). Nanophotonics..

[cit46] Poddubny A., Iorsh I., Belov P., Kivshar Y. (2013). Nat. Photonics.

[cit47] Wang Z. Y., Chen X. M., He X. Q., Fan S. L., Yan W. Z. (2008). Prog. Electromagn. Res..

[cit48] Smolyaninov I. I., Smolyaninova V. (2017). Solid-State Electron..

[cit49] Guo Z., Jiang H., Chen H. (2020). J. Appl. Phys..

[cit50] Bang S., So S., Rho J. (2019). Sci. Rep..

