# Supplementary material for: ZnO–ferromagnetic metal vertically aligned nanocomposite thin films for magnetic, optical and acoustic metamaterials
Source: Nanoscale Adv. 2022 Nov 22;5(1):247–54. doi: 10.1039/d2na00444e (PMC9765661; doi:10.1039/d2na00444e)
Supplement: NA-005-D2NA00444E-s001 [file NA-005-D2NA00444E-s001.pdf]

Electronic Supplementary Material (ESI) for Nanoscale Advances.
This journal is © The Royal Society of Chemistry 2022

Supplemental information:

ZnO-ferromagnetic metal vertically aligned nanocomposite thin films for magnetic, optical and acoustic metamaterials

Robynne L. Paldi[1], Matias Kalaswad[2], Juanjuan Lu[1], James P. Barnard[1], Nicholas A. Richter[1], Mengwei Si[2,3], Nirali A. Bhatt[1], Peide D. Ye[2,3], Raktim Sarma[4], Aleem Siddiqui[4], Jijie Huang[5], Xinghang Zhang, Haiyan Wang*[1,2],

[1] School of Materials Engineering, Purdue University, West Lafayette, Indiana, 47907, USA

[2] School of Electrical and Computer Engineering, Purdue University, West Lafayette, Indiana, 47907, USA

[3] Birck Nanotechnology Center, Purdue University, West Lafayette, 47907, USA

[4] Sandia National Laboratories, New Mexico, USA

[5]School of Materials, Sun Yat-sen University, Guangzhou, Guangdong 510275, China;

* corresponding author: Haiyan Wang, hwang00@purdue.edu

Table S1 The dimensions of metallic nano-inclusions in all films

Material	Height (nm)	Width (nm)
Ni Pillars in ZnO-Ni	18.1 ± 2.2	18.1 ± 5.7
Ni Pillars in ZnO-Ni	27.7 ± 2.2	43.6 ± 1.8
Ni Pillars in ZnO-CoNi	9.8 ± 3.2	8.8 ± 3.9
CoNi Pillars in ZnO-CoNi	28.8 ± 9.9	25.6 ± 13.0
Co Pillars in ZnO-Co	134.0 ± 2.4	31.5 ± 4.6

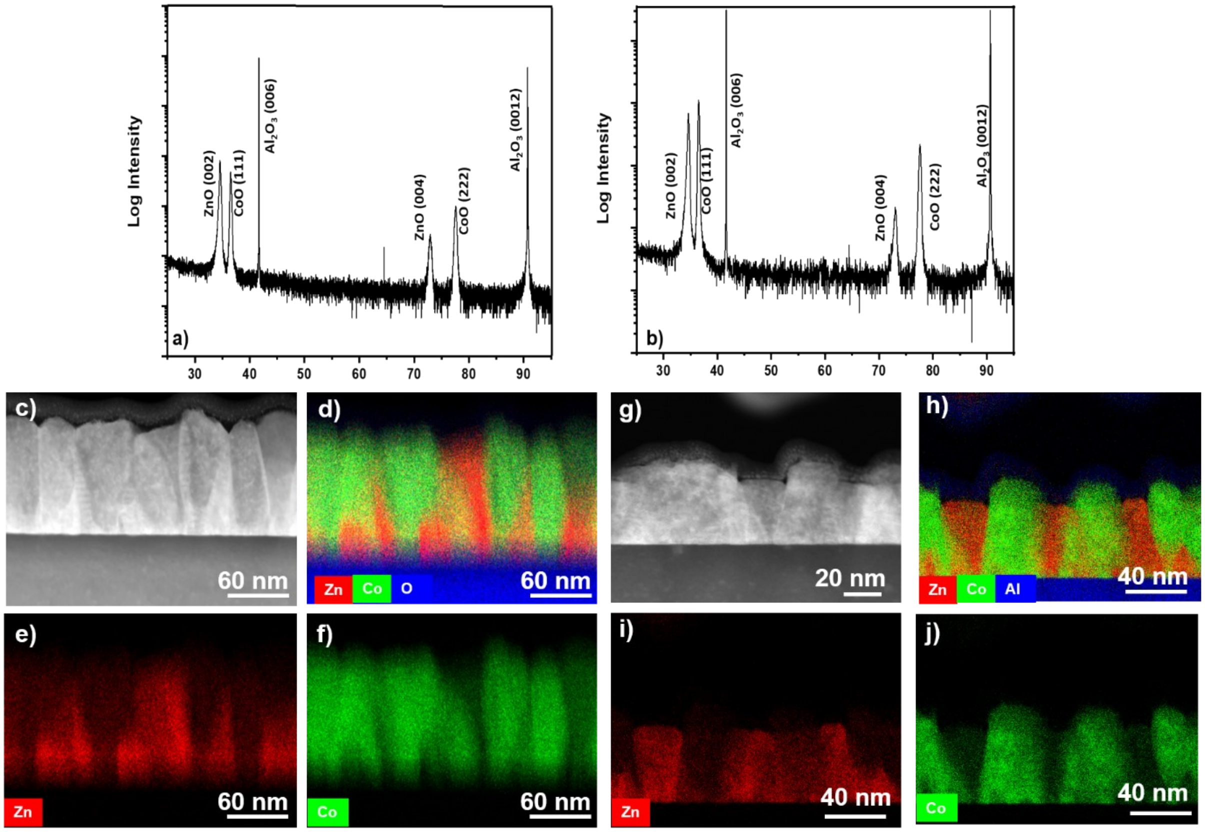

Figure S1. Microstructural characterization of ZnO-Co VAN grown at 500 °C and 700 °C. a) 2θ for 500 °C. b) 2θ XRD scan 700 °C.. c) Cross-section STEM for 500°C. d) Combined EDS-mapping at 500°C. e) EDS-mapping for Zn at 500°C. f) EDS-mapping at 500°C for Co. g) Cross-section STEM at 700°C. h) combined EDS-mapping at 700°C. i) EDS-mapping at 700°C for Zn. j) EDS-mapping at 700°C for Co.

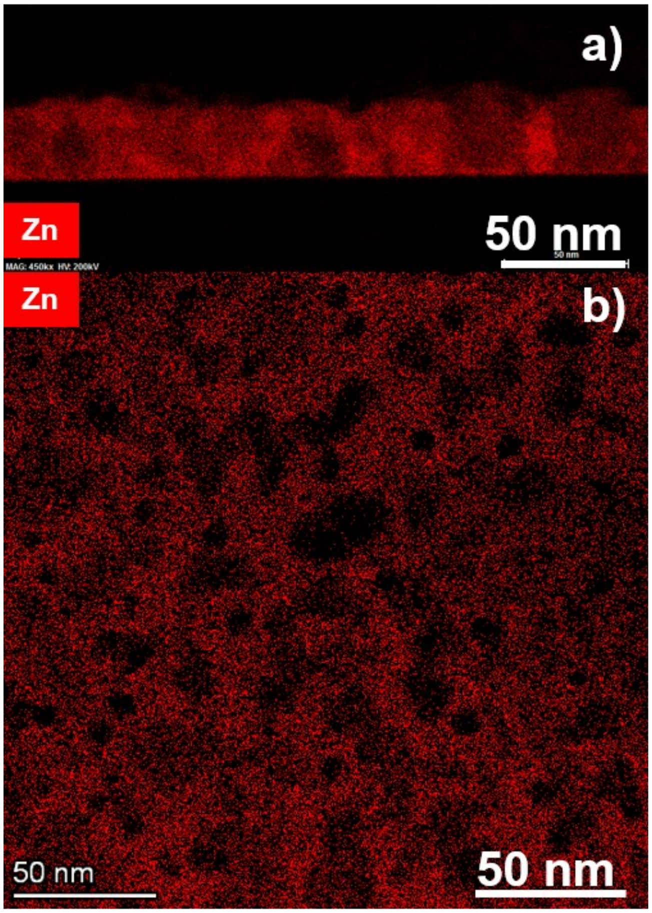

Figure S2 EDS-mapping of Zn in ZnO-Co_xNi_{1-x} VAN. a) Cross-section. b) Plan-view.

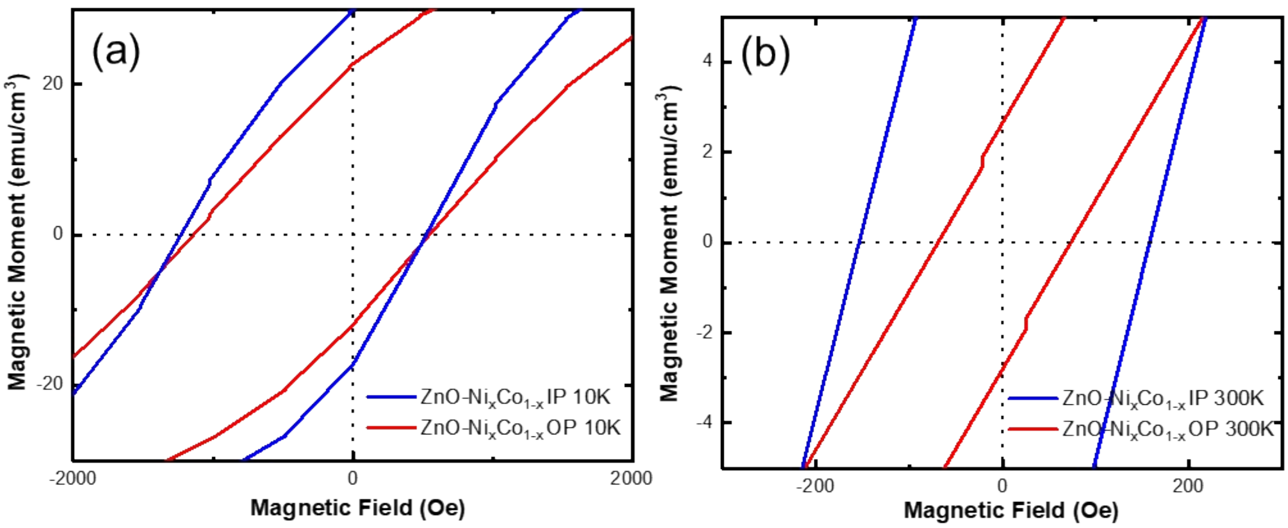

Figure S3 Zoomed features for hysteresis loop of ZnO-Co_xNi_{1-x} at a) 10K and b) 300K.

An enlarged view of the hysteresis loop is shown in Figure S3. The exchange bias indicates the presence of antiferromagnetic domains in the film, which could be the result of the formation of oxide species or Co-doped ZnO. At 10K, the in-plane coercive field H_C is located at -1230.93 and 522.41 O_e while in the out-of plane direction is located at -1140.48 and 544.19 O_e. At 300K, the coercive field for the in-plane direction is -153.71 and 157.84 O_e while the out of plane is -69.19 and 72.98 O_e. The exchange bias is more noticeable at the lower temperature, as the sample is heated it may pass through the Neel temperature of the antiferromagnet present, leading to disordering of the domains and reducing the exchange bias effect. The exchange bias displayed in the ZnO-Co_xNi_{1-x} nanocomposite may lead to applications in giant magnetoresistance (GMR) device and technologies [1].

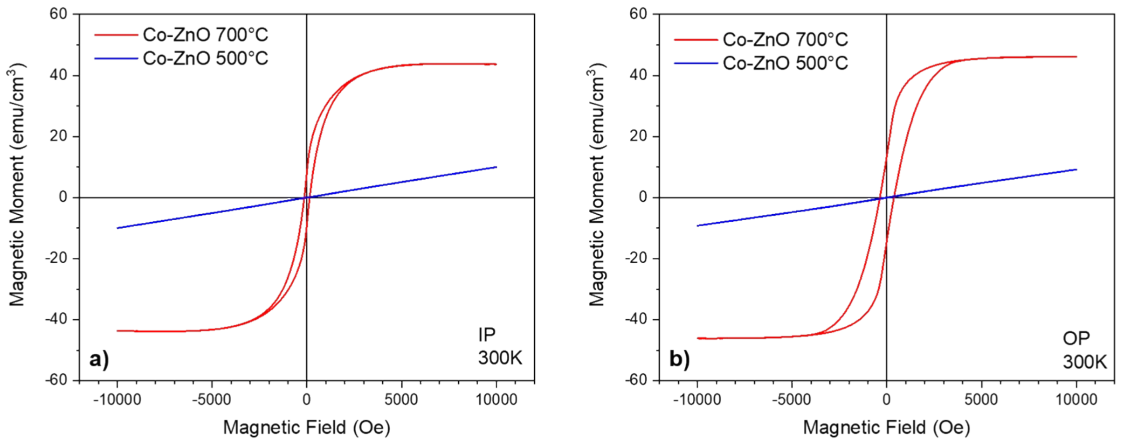

Figure S4 Magnetic measurement for ZnO-Co nanocomposite. a) In-plane and b) out-of plane.

Anisotropic magnetic measurements were also performed at 300K for the ZnO-Co sample in Figure S4 for films grown at 500°C and 700°C. Figure S4a shows in-plane measurement and Figure S4b shows the out-of plane measurement. ZnO-Co demonstrates ferromagnetic response when grown at 700°C and a paramagnetic response when grown at 500°C. The paramagnetic response at 500°C is in line with the microstructure characterization and indicative of the ZnO-CoO oxide-oxide composite growth. When grown at 700°C, the ZnO-Co system demonstrates slightly anisotropic ferromagnetic response, with the coercive field being slightly larger in the out-of plane direction. The induction of ferromagnetic response in the system may be due to small cobalt-rich areas precipitating due to the high growth temperature.

Table S2: Tabulated values for saturation magnetization, coercive field, remnant polarization, and relevant crystallographic direction.

Temperature	Crystallographic Orientation		Coercive Field (Oe)		Magnetization (emu/cc)		
			Negative H_c	Positive H_c	M_s	negative M_r	Positive M_r
**ZnO-$Ni_{66}Co_{33}$**							
300K	IP	$[\bar{1}10]$, $[\bar{1}000]$	-153.71	157.84	51.85	-12.87	12.55
	OP	[111], $[\bar{1}100]$	-69.19	72.98	47.22	-2.81	2.63
10K	IP	$[\bar{1}10]$, $[\bar{1}100]$	-1230.93	522.41	45.34	-17.13	29.97
	OP	[111], $[\bar{1}000]$	-1140.48	544.19	46.39	-12.00	22.78
**ZnO-Ni**							
300K	IP	$[\bar{1}10]$, $[\bar{1}000]$	-184.46	192.36	89.83	-35.77	~~34.64~~
	OP	[111], $[\bar{1}100]$	-58.95	60.76	83.50	-4.45	4.29
10K	IP	$[\bar{1}10]$, $[\bar{1}100]$	-347.26	323.71	84.74	-40.44	41.87
	OP	[111], $[\bar{1}000]$	-340.29	356.54	79.01	--25.71	24.63

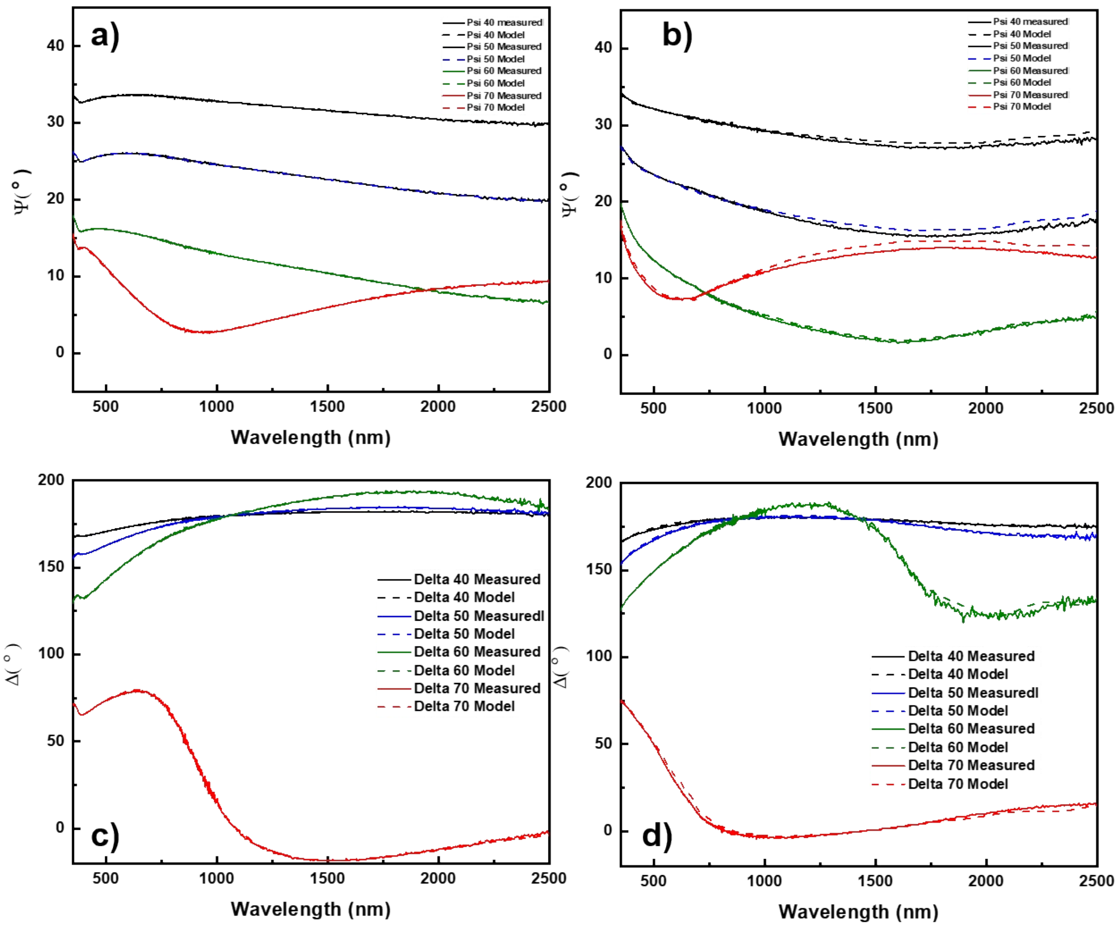

Figure S5. Measured and model psi for a) ZnO-Ni and b) ZnO-Co_xNi_{1-x}. Measured and model delta for c) ZnO-Ni and d) ZnO-Co_xNi_{1-x}.

Table S3 Table 1.1 Sample geometry factor calculated by sample geometry simulator (SGS)

Sample	Geometry Factor	
	IP	OP
ZnO-Ni	0.994	1.165
ZnO-Ni_xCo_{1-x}	0.962	1.174

References:

[1] I. Ennen, D. Kappe, T. Rempel, C. Glenske, A. Hütten, *Sensors (Switzerland)* **2016**, *16*, DOI: 10.3390/s16060904.
